# Association of colorectal adenoma with other malignancies in Swedish families

**DOI:** 10.1038/sj.bjc.6604276

**Published:** 2008-02-19

**Authors:** E Hiripi, J Lorenzo Bermejo, J Sundquist, K Hemminki

**Affiliations:** 1 Division of Molecular Genetic Epidemiology, German Cancer Research Center (DKFZ), Im Neuenheimer Feld 580, 69120, Germany; 2 Karolinska Institute, Center for Family and Community Medicine, Huddinge 141 83, Sweden

**Keywords:** familial risk, colorectal adenoma, extracolorectal cancer

## Abstract

Using the Swedish Family-Cancer Database covering over 11.5 million individuals, estimated relative risks (RRs) for colorectal adenoma were using Poisson's regression. The RR of colorectal adenoma was found to be increased among first-degree relatives of patients with colorectal cancer (2.72; 95% confidence interval=2.46–3.00) and among the offspring and siblings of patients with endometrial and prostate cancers. We also found an increased risk of colorectal adenoma for the offspring of individuals with stomach cancer and leukaemia, and for siblings of those with pancreatic cancer and multiple myeloma. Our results suggest that colorectal adenoma may share a genetic aetiology with cancer even at extracolorectal sites. Increases of colorectal adenoma in families affected by prostate cancer and acute leukaemia cannot be attributed to known cancer syndromes, although the play of chance cannot be excluded.

Most colorectal cancers (CRCs) develop through sequential malignant transformations of adenomatous polyps (Bond, 2000). Mutations in the adenomatous polyposis coli gene are responsible for the early stage of adenoma formation in familial adenomatous polyposis (Houlston, 2001; Lynch and de la Chapelle, 2003; Molatore and Ranzani, 2004) and mutations in the mismatch-repair genes are associated with hereditary nonpolyposis CRC (HNPCC) (Lynch and de la Chapelle, 2003; Molatore and Ranzani, 2004). The later stages of adenoma development into CRCs include K-ras, p53 and mismatch-repair genes (Lynch and de la Chapelle, 2003; Molatore and Ranzani, 2004).

The adenoma–carcinoma sequence implies that colorectal adenomas and carcinomas have common risk factors. A family history of CRC increases the risk of CRC (Slattery and Kerber, 1994; Hemminki and Li, 2001; Andrieu *et al*, 2004; Stefansson *et al*, 2006) and is a strong risk factor for adenoma growth (Lindgren *et al*, 2002; Almendingen *et al*, 2003). Although an association between CRC risk and a family history of extra-colorectal malignancies has been reported previously (Slattery and Kerber, 1994; Hemminki and Li, 2001; Andrieu *et al*, 2004), little is known on the corresponding risk of colorectal adenoma.

We have therefore quantified the risk of colorectal adenoma among individuals with a family history of any cancer using the nationwide Swedish Family-Cancer Database with its unique capacity for accurate and unbiased assessment (Hemminki and Li, 2001; Hemminki *et al*, 2001b).

## MATERIALS AND METHODS

The population-based Swedish Family-Cancer Database was created by linking the Multigeneration Register at Statistics Sweden to the Swedish Cancer Registry (Hemminki *et al*, 2001b). This is based on compulsory reports from pathologists and cytologists, on every cancer diagnosis on surgically removed tissues, biopsies, cytological specimens, bone marrow aspirates and autopsies (Center for Epidemiology, 2004). The Multigeneration Register includes individuals born in Sweden after 1931 and their biological parents and its latest update covers over 11.5 million individuals. Cancer/adenoma data were retrieved from the Swedish Cancer Registry from 1961 to 2004. Database coverage is practically complete but some familial links are missing from offspring born before 1941 and dying in 1960–1997 reducing the number of fatal cancers among offspring. This is unlikely to bias familial studies because familial and sporadic cases would be reduced proportionately (Hemminki *et al*, 1998; Hemminki and Vaittinen, 1999). The present study relies on individuals with information about both parents. Family history was restricted to first-degree relatives, parents, and siblings.

From International Classification of Diseases (ICD-7) the following codes were used: for colorectal adenomas 094; CRCs by anatomic site: 1530 (ascending), 1531 (transverse), 1532 (descending), 1533 (sigmoid), 1538 (multiple sites), 1534 and 1539 (other sites), 154 – excluding 1541 (rectum). Follow-up started from the date of birth, immigration or 1 January 1961, whichever occurred last. Follow-up ended on the date of diagnosis of CRC or colorectal adenoma, death, emigration, or the closing date of the study (31 December 2004), whichever came first. Relative risks (RRs) with 95% confidence intervals (CIs) were used to compare adenoma incidence among those whose family was affected by cancer/adenoma with adenoma incidence in the general population. Cases of adenoma and person – years were classified according to gender, family history of cancer, calendar year and age. The distribution of the number of cases in each group was modelled by Poisson's regression. The Genmod procedure of the SAS software was used for the analysis (SAS Version 9.1; SAS Institute, Cary, NC, USA).

## RESULTS

The Database included 2943 individuals diagnosed with colorectal adenoma and with information about both parents. The parental generation incorporated 12 458 individuals with colorectal adenoma. The incidence of colorectal adenoma in Sweden has been increasing since the 1960s; the site-specific age-adjusted incidence rates for 1990–1999 and 2000–2004 are displayed in Table 1. Table 2 shows some demographic characteristics and RRs. Women were at a slightly lower risk of colorectal adenoma compared to men (RR=0.89; 95% CI=0.82–0.94). As expected, age was associated with risk, which was also increased with a family history of CRC (RR=2.72; 95% CI=2.46–3.00) and particularly of colorectal adenoma (RR=4.99; 95% CI=4.12–6.05). Among the 108 patients with colorectal adenoma and also a family history of this, 55 individuals (in 27 families) had an affected sibling (RR=9.41; 95% CI=7.21–12.3).

Standard Poisson's regression assumes independent observations. Because of possible overdispersion due to clustered family structure, s.e. were adjusted using Pearson's *χ*^2^, divided by the degrees of freedom, resulting in slightly wider CIs. For example, the RR of colorectal adenoma for individuals with a positive family history was 4.99 (95% CI=3.36–7.41). However, as this procedure may be sensitive to outlying observations to be expected in our large data set, we show unadjusted CIs and point out the possibly conservative limits due to familial dependence.

The left column of Table 3 shows colorectal adenoma RRs according to parental history of CRC and colorectal adenoma: it was increased among offspring of patients with colon cancer (RR=2.59; 95% CI=2.28–2.94), rectal cancer (RR=2.59; 95% CI=2.20–3.05), colon adenoma (RR=4.43; 95% CI=3.09–6.34), and rectal adenoma (RR=2.62; 95% CI=1.77–3.89). Corresponding risks with a sibling history of CRC and colorectal adenoma are displayed in the right column of Table 3, with particularly high risks when a sibling had colon (RR=11.3; 95% CI=8.15–15.7) or rectal adenoma (RR=6.94; 95% CI=4.42–10.9). Colorectal adenoma was higher for the offspring of individuals with colon adenoma (RR=4.43) than those with colon cancer (RR=2.59; *P*-value <0.01). It was also higher for those with a sibling with colon adenoma (RR=11.3) than when a sibling had colon cancer (RR=2.64; *P*-value <0.01). Colorectal adenoma risk was higher when a sibling had rectal adenoma (RR=6.94), than when a sibling had rectal cancer (RR=2.75; *P*-value <0.01).

The offspring risk of colorectal adenoma was higher when parents had multiple adenomas than when parents presented with single adenomas (RR=21.6; 95% CI=5.70–82.0). Sibling risk was also significantly higher for multiple than for single adenomas in any colorectal site (RR=20.7; 95% CI=3.48–123) (results not shown).

Table 4 shows colorectal adenoma RRs with a family history of malignancies other than CRC, but only for sites where at least 10 individuals with a family history of cancer had colorectal adenoma.

The risk was increased among the offspring of parents with leukaemia (RR=1.34; 95% CI=1.02–1.76). Among the 53 affected parents, 17 had chronic lymphoblastic, 14 acute myeloid, 5 acute lymphoblastic (ALL), 2 chronic myeloid leukaemias; 7 type unspecified; 8 polycythaemia vera. Colorectal adenoma risk was the highest among the offspring of the five patients with ALL (RR=3.38; 95% CI=1.41–8.14) in which the leukaemias were diagnosed at advanced ages (63–94 years). Risk was also increased among the offspring of parents with acute myeloid leukaemia (RR=1.79; 95% CI=1.06–3.02). It was also increased among individuals with a parental history of stomach (RR=1.28; 95% CI=1.05–1.57), endometrial (RR=1.45; 95% CI=1.10–1.91) and prostate (RR=1.17; 95% CI=1.03–1.34) cancers. Colorectal adenoma showed increases among those whose siblings had multiple myeloma (RR=2.63; 95% CI=1.42–4.90), endometrial (RR=1.63; 95% CI=1.05–2.54) and pancreatic cancer (RR=2.18; 95% CI=1.27–3.77). The risk was also increased when parents or siblings had cancer at any site (RR=1.50; 95% CI=1.39–1.61, RR=1.44; 95% CI=1.30–1.59, respectively).

The risk of site-specific colorectal adenoma was examined according to parental and sibling history of cancer at specific sites (result not shown). Sigmoid adenomas were associated with parental history of liver cancer (RR=1.69; 95% CI=1.09–2.64; *N*=20), and with a sibling history of melanoma (RR=2.06; 95% CI=1.13–3.73; *N*=11). A sibling history of cervical or nervous system cancer was associated with an increased risk of rectal adenoma (RR=2.44; 95% CI=1.31–4.54; *N*=10, RR=1.72; 95% CI=1.02–2.92; *N*=14, respectively). Rectal adenoma was also related to parental history of melanoma (RR=1.63; 95% CI=1.06–2.51 *N*=21).

## DISCUSSION

The present study indicates that individuals with first-degree relatives with colorectal adenoma have an approximately five times higher probability of this neoplasm than those without a family history; the risk being particularly high among relatives of those with multiple adenomas. As in previous studies risk was increased among offspring and siblings of CRC patients (Bonelli *et al*, 1988; Tung and Wu, 2000; Lindgren *et al*, 2002).

To our knowledge, there are no studies of colorectal adenoma in relation to a family history of extracolonic cancers. The present data suggest an involvement of colorectal adenoma in HNPCC and associations with the commonest HNPCC cancer sites – endometrium, stomach, pancreas and nervous system – were identified (Lynch and de la Chapelle, 2003).

We also found colorectal adenoma associated with some extracolorectal cancers unrelated to CRC. There was a higher risk of colorectal adenoma for the offspring and siblings of patients with prostate cancer. The siblings of patients with multiple myeloma and the offspring of patients with leukaemia were at an increased risk of colorectal adenoma. Increased risks of CRC are reported among first-degree relatives of patients with leukaemia (Andrieu *et al*, 2004; Hemminki and Chen, 2004), myeloma (Hemminki and Li, 2001), and prostate cancer (Slattery and Kerber, 1994). We also found that the offspring of those with adult acute lymphoblastic leukaemia were at a higher risk of colorectal adenoma. Rectal adenoma risk was increased among siblings of women with cervical cancer. An increased risk of rectal cancer before the age of 50 years has been reported among the offspring of women with cervical cancer after the age of 50 years (Hemminki and Chen, 2004), but the risk was not increased among siblings of such women. Moreover, the risk of rectal cancer as a second tumour seems to be increased after a first cervical cancer (Hemminki *et al*, 2001a). The familial association of rectal adenoma with melanoma should be investigated using independent data.

Our study had the advantage of information on the anatomical location of the colonic tumours, but our analyses were limited by small numbers, although this problem can be addressed when the Database in further updated. During our study, Sweden had no population-based screening programmes for CRC (Hakama *et al*, 2005). Some individuals in our study may have undergone colonoscopic screening because of a family history of CRC, as the risks reported in Tables 2 and 3 could be overestimated. Another limitation is that only precancerous lesions and gastrointestinal polyps with suspected malignancy are compulsorily reported to the Cancer Registry and some adenomas might be already precancerous. A shortcoming is the lack of data on tobacco smoking, alcohol consumption, or diet.

In conclusion, a family history of colorectal adenoma is a risk factor for colorectal adenoma particularly for first-degree relatives of patients with multiple adenomas. Unrelated to known CRC syndromes, we found increases among the offspring and siblings of patients with prostate cancer, and the offspring of patients with leukaemia. However, given the number of tests performed, chance may have operated in some of our findings. Our data may help to understand the adenoma–carcinoma sequence and to develop prevention strategies.

## Figures and Tables

**Table 1:** Age standardised incidence of colorectal adenoma/100 000 person-years diagnosed in Sweden between 1990 and 2004 (adjusted to world standard population 2000)

**Site**	**1990–1999**	**2000–2004**
Colorectum	3.29	5.03
Colon	1.65	2.89
Ascending	0.24	0.52
Transverse	0.15	0.31
Descending	0.07	0.16
Sigmoid	0.81	1.34
Other	0.36	0.52
Multiple	0.02	0.04
Rectum	1.64	2.14

**Table 2:** Relative risk of colorectal adenoma according to sex, age, calendar year, and family history of colorectal cancer/adenoma

**Covariate**	**Level**	***N***	**RR (95% CI)**
Gender	Females	1365	**0.89** (0.82–0.94)
	Males	1578	1.00 (ref.)
			
Age of diagnosis	Before 50	706	**0.03** (0.02–0.04)
	50–54	449	**0.26** (0.21–0.33)
	55–59	628	**0.49** (0.40–0.59)
	60 and above	1160	1.00 (ref)
			
Calendar year of diagnosis	Before 1980	90	**0.18** (0.11–0.28)
	1980–1989	282	**0.58** (0.43–0.78)
	1990–1999	1038	**0.67** (0.57–0.79)
	2000–2004	1533	1.00 (ref.)
			
Family history of colorectal cancer	Yes	474	**2.72** (2.46–3.00)
	No	2469	1.00 (ref.)
			
Family history of colorectal adenoma	Yes	108	**4.99** (4.12–6.05)
	No	2835	1.00 (ref.)

Bold signifies *P*<0.05.

**Table 3:** Relative risk of colorectal adenoma for the offspring/sibling of individuals with colorectal cancer/adenoma

	**Colorectal adenoma**	
	**Offspring**	**Sibling**
	***N***	***N***
**Location in parent/sibling**	**RR (95% CI)**	**RR (95% CI)**
*Invasive cancer*		
Colon	264	50
	**2.59** (2.28–2.94)	**2.64** (2.00–3.50)
		
Rectum	153	33
	**2.59** (2.20–3.05)	**2.75** (1.95–3.88)
		
*Adenoma*		
Colon	30	36
	**4.43** (3.09–6.34)	**11.3** (8.15–15.7)
		
Rectum	25	19
	**2.62** (1.77–3.89)	**6.94** (4.42–10.9)

Bold signifies *P*<0.05.

**Table 4:** Relative risk of colorectal adenoma for the offspring/siblings of individuals with invasive cancer

	**Colorectal adenoma**	
	**Offspring**	**Sibling**
	***N***	***N***
**Cancer site in parent/sibling**	**RR (95% CI)**	**RR (95% CI)**
Tongue/mouth	27	5
	1.12 (0.76–1.63)	0.79 (0.33–1.89)
		
Stomach	100	9
	**1.28** (1.05–1.57)	1.35 (0.70–2.59)
		
Liver	56	6
	1.22 (0.93–1.58)	1.12 (0.50–2.49)
		
Pancreas	51	13
	1.12 (0.85–1.48)	**2.18** (1.27–3.77)
		
Lung	108	31
	1.13 (0.93–1.37)	1.35 (0.95–1.92)
		
Breast	151	80
	1.04 (0.88–1.22)	1.05 (0.84–1.31)
		
Cervix uteri	22	13
	0.81 (0.53–1.23)	1.46 (0.85–2.51)
		
Endometrium	51	20
	**1.45** (1.10–1.91)	**1.63** (1.05–2.54)
		
Ovary	28	11
	0.88 (0.60–1.27)	0.93 (0.52–1.69)
		
Prostate	233	59
	**1.17** (1.03–1.34)	**1.45** (1.12–1.88)
		
Kidney	55	4
	1.22 (0.93–1.59)	**3.25** (1.22–8.68)
		
Urinary organs	72	12
	1.10 (0.87–1.39)	0.87 (0.49–1.53)
		
Melanoma	36	28
	1.30 (0.94–1.81)	1.34 (0.93–1.95)
		
Squamous cell skin	51	13
	0.94 (0.71–1.24)	1.67 (0.97–2.87)
		
Nervous system	37	21
	1.06 (0.77–1.47)	1.19 (0.77–1.83)
		
Endocrine glands	18	13
	0.88 (0.55–1.40)	1.36 (0.79–2.34)
		
Connective tissue	10	
	1.16 (0.62–2.15)	
		
Non-Hodgkin lymphoma	39	3
	1.04 (0.76–1.43)	1.26 (0.41–3.91)
		
Multiple myeloma	21	10
	0.93 (0.61–1.44)	**2.63** (1.42–4.90)
		
Leukaemia	53	9
	**1.34** (1.02–1.76)	0.95 (0.49–1.83)
		
Any cancer	1446	451
	**1.50** (1.39–1.61)	**1.44** (1.30–1.59)

Bold signifies *P*<0.05.
